# Plant-Based Diets as a Therapeutic Strategy for Obesity: Modulation of Adipogenesis, Inflammation, Energy Homeostasis, and Metabolic Dysfunction

**DOI:** 10.3390/nu18213592

**Published:** 2026-10-29

**Authors:** Hanyue Shi, Jared Sieusahai, Marina Kamel, Clarence Wong, Andrew Mason, Zobair Younossi, Christophe Corpechot, Eduardo Grunvald, Olexandr Troshyn, Andrea Galli, Sahar Iqbal, Jasdeep Saluja, Sebastian Straube, Ellina Lytvyak

**Affiliations:** 1 Division of Preventive Medicine, Department of Medicine, Faculty of Medicine & Dentistry, University of Alberta, Edmonton, AB T6G 2T4, Canada; hanyue11@ualberta.ca (H.S.); jsieusah@ualberta.ca (J.S.);; 2 Department of Medicine, University of Galway, H91 TK33 Galway, Ireland; 3 Division of Gastroenterology, Department of Medicine, University of Alberta, Edmonton, AB T6G 2X8, Canada; 4 Global NASH/MASH Council Center for Outcomes Research in Liver Disease, Washington, DC 20037, USA; 5 Global Center for Liver Outcomes & Policy Research, School of Medicine, Georgetown University, Washington, DC 20057, USA; 6 Hôpital Saint-Antoine, Sorbonne University, 75012 Paris, France; 7 Division of General Internal Medicine, Department of Medicine, University of California San Diego, La Jolla, CA 92093, USA; 8 Department of Medicine, University of Alberta, Edmonton, AB T6G 2B7, Canada; 9 Gastroenterology Research Unit, Department of Experimental and Clinical Biomedical Sciences “Mario Serio”, University of Florence, 50134 Florence, Italy; 10 Faculty of Medicine, Memorial University of Newfoundland, St. John’s, NL A1B 3V6, Canada; 11 Aroga Lifestyle Medicine, Victoria, BC V8Z 5G5, Canada

**Keywords:** obesity, plant-based diet, adipose tissue, gut microbiota, short-chain fatty acids, insulin resistance, chronic inflammation, lipid metabolism, dietary fibre, cardiometabolic health

## Abstract

**Background and Objectives:** Obesity is a chronic, relapsing disease driven by dysregulated energy balance, appetite, adipose tissue function, and metabolic homeostasis. Dietary modification remains central to long-term management. Plant-based diets (PBDs), particularly those emphasizing minimally processed plant foods, are associated with lower adiposity and improved cardiometabolic health. This review synthesizes human evidence on weight-loss efficacy and mechanisms linking PBDs to obesity and adiposity-related outcomes. **Methods:** Following PRISMA-ScR guidance, PubMed and Scopus were searched for English-language adult human studies published from January 2010 to August 2026. Of 1439 records identified and 1297 screened after deduplication, 168 studies met the inclusion criteria and were synthesized descriptively. **Results:** Human studies support beneficial effects of PBDs on spontaneous energy intake, insulin sensitivity, visceral and ectopic lipid accumulation, and circulating lipid profiles. Clinical trials indicate that PBDs can support weight loss, although superiority over other dietary approaches needs further exploration. Proposed mechanisms include lower dietary energy density and greater satiation, improved lipid partitioning, increased postprandial energy expenditure, reduced oxidative and inflammatory stress, and altered gut microbial metabolism and enteroendocrine signaling. Evidence also suggests diet-responsive epigenetic changes. Direct human evidence for effects on central appetite circuitry, brown or beige adipose tissue, and weight-independent molecular pathways remains limited. **Conclusions:** High-quality PBDs represent a biologically coherent and scalable strategy for obesity management, with evidence suggesting benefits for energy intake and selected aspects of cardiometabolic health, including energy intake, insulin resistance, lipid metabolism, systemic inflammation, and gut-derived signaling. These effects position high-quality PBDs as a promising strategy for reducing the risk of obesity-related complications, including type 2 diabetes, cardiovascular disease, MASLD, and selected cancers. Further well-designed controlled trials will help clarify the independent contributions of dietary composition and the beneficial effects mediated through reduced energy intake and sustained weight loss.

## 1. Introduction

Globally, the number of adults with overweight or obesity more than doubled between 1990 and 2021, reaching approximately 2.11 billion in 2021, while the number of children and adolescents affected rose from 198 million to 493 million over the same period [1,2]. If current trends continue, an estimated 3.80 billion adults and 746 million children and adolescents are projected to be living with overweight or obesity by 2050 [1,2].

Once concentrated in high-income countries, obesity is now rising most rapidly in Asia and Africa, with the number of affected adults in the sub-Saharan Africa super-region alone forecast to increase by 255% by 2050, compounding rather than replacing the persisting burden of undernutrition [1]. This burden is not confined to any one region or income level. In Canada, national survey data show that adult obesity prevalence increased significantly from 22.2% to 27.2% between 2005 and 2018 [3].

The clinical consequences of obesity extend across nearly every organ and system. Obesity is one of the principal drivers of type 2 diabetes (T2D), cardiovascular disease (CVD), dyslipidemia, several cancers, osteoarthritis, headache disorders, and metabolic dysfunction-associated steatotic liver disease (MASLD) [4,5,6,7,8,9,10,11,12]. Notably, the global age-standardised burden attributable to high body mass index (BMI) increased between 2010 and 2023, with attributable DALY rates rising by 10.5% over this period [6]. The associated burden of individual conditions is substantial: cancers attributable to high BMI represent a growing share of the global cancer burden, and MASLD, now the leading cause of chronic liver disease, affects an estimated 1.3 billion individuals worldwide, projected to reach 1.8 billion by 2050 [8,9]. The liver is a particularly sensitive target of excess adiposity: MASLD is present in the majority of individuals with obesity, and its progressive form, metabolic dysfunction-associated steatohepatitis (MASH), can advance to fibrosis, cirrhosis, and hepatocellular carcinoma, making it a rapidly growing indication for liver transplantation [13,14,15]. Because hepatic steatosis is driven by the same sustained positive energy balance, insulin resistance (IR), and adipose tissue dysfunction that underlie obesity, MASLD is increasingly regarded as the hepatic manifestation of the metabolic syndrome, and weight reduction remains the cornerstone of its management [13,14,15].

The economic dimension is comparably severe: obesity-related healthcare costs and productivity losses consumed an estimated 2.19% of global gross domestic product in 2019, a figure projected to reach 3.29% by 2060 if current trends continue [16]. Part of this burden reflects obesity’s impact on employment and work capacity, an important social determinant of health, with unemployment consistently associated with poorer physical and mental health outcomes [17]. Moreover, this escalating burden may outpace the healthcare resources available to address it, as global analyses project persistent shortfalls in the health workforce required to meet population health needs [18].

Obesity is now understood not as a disorder of individual willpower but as a chronic, relapsing disease arising from complex interactions among genetic, behavioural, environmental, and socioeconomic factors, in which dysfunctional adipose tissue expansion and a sustained positive energy balance drive progressive metabolic deterioration [19]. This deterioration is closely mirrored in the liver, where ectopic lipid accumulation, lipotoxicity, and hepatic IR establish a self-reinforcing cycle between adiposity and liver injury [20].

Against this burden, pharmacological and surgical therapies have advanced considerably, most recently through glucagon-like peptide 1 (GLP-1) and gastric inhibitory polypeptide (GIP) receptor agonists, yet important limitations persist [21,22]. These agents, while effective, might not be available or accessible for some patients and are associated with weight regain on discontinuation [23,24]. Bariatric surgery, though also effective for some patients, is resource-intensive, has a substantial risk of weight regain, and may carry short- and long-term post-surgical risks [21]. Pharmacotherapy for MASLD itself remains limited, with few approved liver-directed agents, so lifestyle intervention continues to be recommended as first-line therapy in current hepatology guidelines; a sustained weight loss of approximately 5–10% is associated with reductions in hepatic steatosis and, at the higher end, with resolution of steatohepatitis and regression of fibrosis [15]. Consequently, dietary modification remains an indispensable and enduring foundation of obesity and MASLD management, retaining relevance across the full spectrum of disease severity and providing a foundation on which pharmacological and surgical interventions are layered rather than a strategy they replace [15].

Among dietary approaches, plant-based diets (PBDs) have recently attracted particular interest. Characterized by a high intake of vegetables, fruits, legumes, whole grains, nuts, and unsaturated plant-derived fats, with reduced consumption of animal products, these patterns are consistently associated with lower adiposity, improved cardiometabolic risk profiles, and favourable metabolic health [25,26]. Observational cohorts and intervention studies further associate healthful PBDs with lower prevalence of MASLD, reduced hepatic fat content, and improved markers of liver injury, independent of, and in addition to, their effects on body weight [27,28,29,30]. Their effects appear to arise not from any single constituent but from the combined action of a fibre-rich, polyphenol-dense food matrix of low energy density that engages numerous pathways governing energy homeostasis and adipose biology, as well as hepatic handling and inflammation [31,32]. Crucially, the quality of plant-based intake is itself a determinant of benefit, since patterns emphasizing refined grains and sugar-sweetened products may confer little advantage or even harm, whereas those built on minimally processed whole plant foods are the ones associated with improved outcomes [25]. This distinction is especially pertinent to the liver, given that dietary fructose and refined carbohydrates are potent stimuli of hepatic de novo lipogenesis (DNL) [33,34]. Throughout this review, a distinction is drawn between healthful PBDs, which emphasize whole or minimally processed plant foods such as vegetables, fruits, legumes, whole grains, and nuts, and unhealthful PBDs, which are higher in refined grains, sugar-sweetened beverages, and processed plant foods; this distinction is central to interpreting their metabolic and hepatic effects [25]. Beyond their effects on body weight, PBDs and their constituents act upon multiple pathogenic mechanisms underlying obesity and MASLD, including appetite regulation and energy intake, insulin sensitivity, gut microbial metabolism, adipose tissue inflammation, oxidative stress, lipid metabolism, hepatic DNL and fat oxidation, the gut–liver axis and epigenetic regulation [32]. Evaluating clinical weight-loss evidence alongside the cellular and molecular mechanisms relevant to PBDs in obesity management is the aim of this comprehensive review. The dietary profiles discussed are defined in Table 1.

## 2. Materials and Methods

### 2.1. Data Sources and Search Strategy

This review examines the cellular and molecular mechanisms through which PBDs influence obesity and related metabolic dysfunction, with an emphasis on human studies. A structured literature search and search strategy were designed to identify evidence examining the effect of PBDs on obesity and adiposity-related outcomes across main mechanistic domains. Searches were performed in PubMed (National Library of Medicine) and Scopus (Elsevier). The review was designed and reported in accordance with the Preferred Reporting Items for Systematic Reviews and Meta-Analyses extension for Scoping Reviews (PRISMA-ScR).

For each mechanistic domain, search strings combined terms for the PBDs’ exposure with domain-specific terms for the outcome of interest (for example, appetite regulation with satiety; insulin sensitivity and glucose metabolism; gut microbiota and the gut–brain axis; adipose tissue biology and adipogenesis; chronic inflammation; oxidative stress; energy expenditure and thermogenesis; and lipid metabolism) and with terms restricting results to human studies.

Controlled vocabulary and free-text terms were combined across two principal concepts: obesity/adiposity and PBDs. The obesity domain incorporated the MeSH descriptors “Obesity” (Tree Numbers: C18.654.726.750.500 and C23.888.144.699.500; MeSH Unique ID: D009765) and “Obesity, Abdominal” (Tree Numbers: C18.654.726.750.500.615 and C23.888.144.699.500.063; MeSH Unique ID: D056128), together with the latter’s entry terms: “Obesity,” “Abdominal Obesities,” “Obesities, Abdominal,” “Abdominal Obesity,” “Central Obesity,” “Central Obesities,” “Obesities, Central,” “Obesity, Central,” “Obesity, Visceral,” “Visceral Obesity,” “Obesities, Visceral,” and “Visceral Obesities.” Free-text terms addressing obesity, abdominal/central obesity, visceral adiposity, and related anthropometric outcomes were additionally incorporated. The dietary domain comprised terms including, but not limited to, “plant-based diet,” “plant-based diets,” “plant-based dietary pattern(s),” “whole-food plant-based,” “plant-based dietary index,” “vegetarian,” “vegan.” The two conceptual domains were combined using Boolean operators, with database-specific adaptations to indexing terminology, field restrictions, truncation, and syntax.

Searches were limited to English-language journal articles published from 1 January 2010 through the final search date in August 2026. Results were restricted to human studies. Targeted contextual literature checks during revision were considered separately from the original search and study-selection counts.

### 2.2. Eligibility Criteria

Studies were eligible if they enrolled human adults aged 18 years or older with overweight, obesity, or related metabolic dysfunction, or provided adult human mechanistic evidence directly relevant to the review’s domains; evaluated a defined PBD exposure or intervention (vegan, vegetarian, whole-food plant-based, predominantly plant-based, or a validated PBD index); and reported at least one relevant cellular, molecular, metabolic, or physiological outcome. Eligible designs comprised peer-reviewed primary empirical studies, including randomized and non-randomized interventions, prospective and retrospective cohorts, cross-sectional studies, and case–control studies. Isolated supplements, single nutrients, and single plant foods were not treated as direct whole-diet evidence unless the study also evaluated a defined PBD contrast; such reports were retained only as indirect mechanistic support where relevant.

### 2.3. Exclusion Criteria

Studies were excluded if they were limited to in vitro evidence; enrolled pediatric, pregnancy or lactation, or type 1 diabetes populations. Studies were also excluded if they were listed as reviews, protocols, editorials, commentaries, conference records, or case reports; lacked a defined PBD exposure; or did not report a relevant mechanism or outcome. These exclusions apply to the primary human synthesis; experimental longevity studies are discussed separately as context in Section 4.7.

### 2.4. Study Selection Process

**Figure 1:**
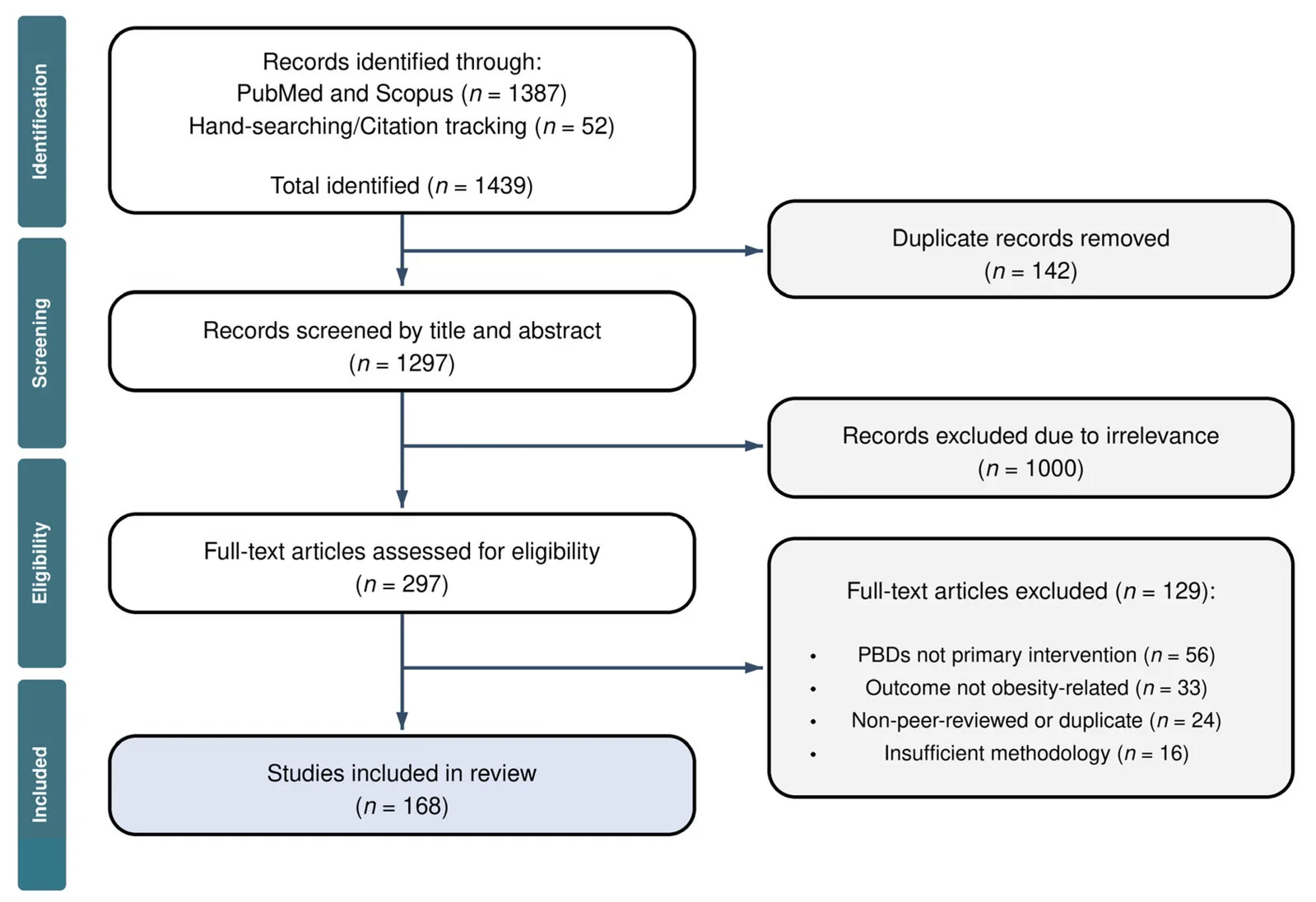
PRISMA-ScR flow diagram of study identification and selection. The electronic search of PubMed and Scopus yielded 1387 records, with a further 52 records identified through hand-searching and citation tracking (total identified, *n* = 1439). After removal of 142 duplicates, 1297 records proceeded to title and abstract screening, of which 1000 were excluded as not relevant to the review question. 297 reports were subsequently assessed in full text. Of these, 129 were excluded: 56 did not evaluate PBDs as the primary intervention, 33 did not report obesity-related outcomes, 24 were non-peer-reviewed publications or duplicates, and 16 provided insufficient methodological information to determine eligibility. The final review therefore comprised 168 studies. Dark blue labels identify the stages of study selection; grey boxes indicate removed or excluded records, and the light blue box indicates studies included in the review. *n* denotes the number of records, reports, or studies at the indicated stage.

Two researchers independently performed the literature search and study selection. Records retrieved from academic databases were combined with reports identified through hand-searching the reference lists of included studies, then de-duplicated. Titles and abstracts were screened against the eligibility criteria, and the full texts of potentially eligible reports were retrieved and assessed. For each excluded full text, a primary reason for exclusion was recorded. The electronic search of PubMed and Scopus yielded 1387 records, with a further 52 records identified through hand-searching and citation tracking (total identified, *n* = 1439). After removal of 142 duplicates, 1297 records proceeded to title and abstract screening, of which 1000 were excluded as not relevant to the review question. 297 reports were subsequently assessed in full text. Of these, 129 were excluded: 56 did not evaluate PBDs as the primary intervention, 33 did not report obesity-related outcomes, 24 were non-peer-reviewed publications or duplicates, and 16 provided insufficient methodological information to determine eligibility. The final review therefore comprised 168 studies. Any discrepancies arising during the screening and eligibility assessment processes were resolved through discussion and consensus. The selection process is summarized in the PRISMA-ScR flow diagram (Figure 1).

### 2.5. Evidence Synthesis

Given the mechanistic and conceptual breadth of the included literature and heterogeneity of study designs, exposures, and outcomes, a narrative synthesis was undertaken, organized by mechanistic domain. Throughout, a distinction is maintained between evidence derived from whole PBDs and evidence derived from isolated dietary components or from preclinical models, and the strength and directness of the supporting human evidence is indicated within each domain. Consistent with scoping-review methodology, findings were charted descriptively rather than pooled statistically, and no formal risk-of-bias appraisal was undertaken.

## 3. Results

Clinical weight-loss findings provide the context for the mechanisms below. In 244 adults, a 16-week low-fat vegan diet reduced weight by 6.4 kg versus 0.5 kg with no dietary change (between-group effect, −5.9 kg) [29]. In a crossover trial of 62 adults, 16-week phases produced changes of −6.0 kg with a low-fat vegan diet and 0.0 kg with a Mediterranean diet [41]. However, the NEW Soul trial in 159 African American adults found no significant difference at 12 months between vegan and low-fat omnivorous diets with comparable nutrition education (−2.39 versus −2.03 kg; *p* = 0.64) [42]. Thus, PBDs have been shown to support weight loss; however, the magnitude varies with the dietary prescription, comparator, and adherence, and greater weight loss than with other healthful diets is not consistently demonstrated.

### 3.1. Energy Balance, Appetite Regulation and Satiety

Energy balance is regulated through interactions among homeostatic, reward-related, cognitive, and peripheral signals [43]. Hunger promotes food seeking, satiation determines when a meal ends, and satiety reduces eating between meals. Minimally processed PBDs can influence these processes through their fibre and water content, low energy density, gastrointestinal effects, and production of microbial metabolites [44,45]. Human evidence is strongest for reductions in spontaneous energy intake and changes in peripheral satiation. Direct effects of PBDs on central appetite circuits remain largely underexplored [45,46].

#### 3.1.1. Neuroendocrine Integration of Appetite and Energy Balance

The hypothalamus integrates signals related to adiposity, nutrient availability, and recent food intake [43]. Within the arcuate nucleus, neuropeptide Y/agouti-related peptide (NPY/AgRP) neurons promote feeding, whereas pro-opiomelanocortin/cocaine- and amphetamine-regulated transcript (POMC/CART) neurons activate anorexigenic melanocortin pathways [47,48]. Leptin and insulin generally favour anorexigenic signaling, while ghrelin promotes hunger. This canonical framework, summarized by Morton et al., was largely established through experimental physiology and provides a mechanistic basis for interpreting human appetite regulation [49].

Hypothalamic regulation is also shaped during early development. Maternal obesity has been linked to altered hypothalamic programming in offspring, potentially affecting appetite and obesity susceptibility later in life [50]. Much of this evidence is preclinical, however, and it does not show that PBDs can reverse developmental changes in hypothalamic function.

Homeostatic regulation interacts with mesolimbic reward pathways and prefrontal systems involved in dietary decision-making and inhibitory control [51,52]. This interaction allows highly palatable foods to promote eating beyond immediate energy requirements [51,52]. Insulin provides one connection between metabolic state and food reward: intranasal insulin reduced food palatability and mesolimbic food-value signals in insulin-sensitive adults, whereas these responses were diminished in participants with insulin resistance [53]. Direct human evidence that PBDs modify hypothalamic, mesolimbic, or prefrontal signaling is still lacking.

#### 3.1.2. Peripheral Hormonal Signals of Hunger, Satiation, and Satiety

Leptin and insulin provide longer-term information about energy availability [49]. Both hormones are commonly elevated in obesity, but reduced central sensitivity can weaken their appetite-suppressing effects [49]. Obesity therefore involves sustained resistance to these metabolic signals rather than absolute hormone deficiency [54].

Meal-related signals operate over shorter timescales. Cholecystokinin (CCK) acts rapidly during eating, whereas GLP-1, GIP, peptide YY (PYY), and pancreatic amylin contribute to meal termination and postprandial satiety [55]. Ghrelin, secreted by the gastric mucosa, primarily promotes hunger [56]. The physiological role of CCK is supported by human receptor-blockade studies in which inhibition of the CCK1 receptor increased caloric intake and hunger and delayed fullness [57].

GIP functions mainly as an incretin by enhancing glucose-dependent insulin secretion [58]. In healthy adults, exogenous GIP altered glucose-insulin handling and accelerated gastric emptying without significantly changing hunger or satiety. GLP-1, by comparison, delayed gastric emptying and reduced several appetite measures [59]. Pharmacological glucagon infusion has also reduced subsequent food intake, although the role of normal postprandial glucagon secretion in appetite regulation remains uncertain [60]. These hormones should therefore not be treated as equivalent satiety signals. Evidence is stronger for GLP-1, PYY, CCK, and amylin than for GIP or glucagon [55]. In dietary studies, changes in circulating hormone concentrations are most informative when accompanied by measurements of subjective appetite, gastric emptying, or subsequent energy intake.

#### 3.1.3. Plant-Based Dietary Modulation of Satiation and Satiety

The physical properties of minimally processed plant foods are central to their effects on energy intake. Vegetables, fruits, legumes, and whole grains generally provide more fibre and water and less energy per unit weight than many animal-based or highly processed foods [61]. Their greater volume promotes gastric distension, while chewing and oral processing allow satiation signals to develop before substantial energy is consumed. Soluble and viscous fibres also delay gastric emptying and nutrient absorption [61,62]. These effects depend on food structure and cannot be assumed for ultra-processed plant-based products [61,62].

A randomized crossover study compared a tofu-based meal with an energy- and macronutrient-matched meal containing processed meat and cheese in males with T2D, obesity, or normal weight [46]. The plant-based meal produced greater subjective satiety and increased postprandial amylin across all three groups. GLP-1 and PYY responses varied according to metabolic status [46]. As the meals also differed in fibre, saturated fat, and beverage composition, the results reflect the overall meal rather than animal-food exclusion alone.

Fermentable fibre also influences appetite through microbial short-chain fatty acids (SCFAs). Targeted colonic propionate delivery increased PYY and GLP-1 concentrations and reduced subsequent energy intake; over 24 weeks, inulin–propionate ester supplementation also limited substantial weight gain and abdominal fat accumulation in adults with overweight [44]. These findings support an appetite-related role for microbial metabolites, but targeted propionate delivery is not equivalent to consuming a whole PBD. The broader microbiota mechanism is discussed below.

Energy density has more direct whole-diet evidence. In a controlled inpatient crossover trial, energy intake was approximately 2.88 MJ/day (689 kcal/day) lower during a minimally processed, low-fat PBD than during an animal-based ketogenic diet, despite similar ratings of hunger, fullness, and meal satisfaction [45]. The PBD provided more fibre and less energy per unit weight, allowing participants to consume a greater food volume relative to its energy content. Although differences in fat, carbohydrate, and food source prevent attribution to a single component, the trial shows that low-energy-density PBDs can reduce spontaneous intake without increasing perceived hunger.

The evidence is more conclusive for food structure, fibre fermentation, and energy density than for uniform changes in individual appetite hormones. Future whole-diet trials should combine ad libitum food-intake measurements with assessments of gastric function, microbial metabolites, and appetite hormones to identify which responses directly mediate reduced energy intake.

#### 3.1.4. Sleep as a Modifier of Appetite Regulation

Sleep duration also modifies energy balance. In adults with overweight who habitually slept for less than 6.5 h per night, extending sleep by approximately 1.2 h per night reduced objectively measured energy intake by about 1.13 MJ/day (270 kcal/day) [63]. Conversely, reducing habitual sleep by approximately 78 min per night for six weeks resulted in modest weight gain and increased sedentary time [64].

Evidence connecting PBDs with sleep is less developed. Greater adherence to a healthful PBD index has been associated with better sleep quality, whereas unhealthy PBDs have been associated with poorer sleep [65]. As these findings are observational, sleep is best considered a modifying factor rather than an established mechanism through which PBDs regulate appetite.

### 3.2. Insulin Sensitivity and Glucose Metabolism

#### 3.2.1. Insulin Signaling and Obesity-Related Insulin Resistance

Beyond its central role in appetite regulation, insulin coordinates postprandial glucose metabolism across skeletal muscle, liver, and adipose tissue [66]. In skeletal muscle, activation of the insulin receptor and insulin receptor substrate proteins initiates phosphoinositide 3-kinase/protein kinase B (PI3K/Akt) signaling, promoting translocation of glucose transporter type 4 (GLUT4) to the cell membrane and increasing glucose uptake [66]. In the liver, insulin suppresses endogenous glucose production and promotes glycogen synthesis, while in adipose tissue it restrains lipolysis and the release of non-esterified fatty acids [66]. Together, these actions maintain glucose homeostasis after nutrient intake [66].

In obesity, chronic nutrient excess and adipose tissue dysfunction increase fatty acid delivery to the liver and skeletal muscle. Accumulation of bioactive lipid intermediates, including diacylglycerols and ceramides, can interfere with insulin receptor and IRS–PI3K/Akt signaling, thereby impairing insulin-stimulated muscle glucose uptake and weakening the suppression of hepatic glucose production [66,67]. IR in adipose tissue further increases fatty acid and glycerol flux to the liver, reinforcing ectopic lipid accumulation and hepatic gluconeogenesis [66,67]. Although these pathways provide a strong mechanistic framework, direct human evidence that PBDs restore individual components of the IRS-PI3K/Akt-GLUT4 pathway remains limited.

#### 3.2.2. Effects of Plant-Based Diets on Insulin Sensitivity

Randomized trials generally support a beneficial effect of PBDs on systemic markers of insulin sensitivity, particularly in adults with overweight or obesity. A meta-analysis of eight trials involving 716 participants found that vegan and vegetarian diets reduce the homeostatic model assessment of insulin resistance (HOMA-IR) and fasting insulin relative to control diets in this population [68]. Evidence was less consistent in participants with prediabetes or T2D, and most studies relied on fasting surrogate markers rather than direct measurements of tissue-specific insulin action.

The most detailed whole-diet evidence comes from a 16-week randomized trial of 244 adults with overweight or obesity. An ad libitum low-fat vegan diet produced a between-group treatment effect of −5.9 kg, decreased HOMA-IR, and improved predicted insulin sensitivity compared with no dietary change. In a magnetic resonance spectroscopy substudy of 44 participants, hepatocellular and intramyocellular lipid levels decreased by 34.4% and 10.4%, respectively, and changes in both lipid compartments were associated with improvements in IR [29]. Baseline hepatocellular lipid content in this subset was generally within the normal range, limiting extrapolation of these findings to individuals with established MASLD [29]. These findings support ectopic lipid reduction as a potential mediator linking low-fat vegan diets with improved insulin sensitivity. However, the intervention also reduced spontaneous energy intake, body weight, and fat mass; consequently, the effects of dietary composition cannot be separated from those of weight loss.

An active-comparator crossover trial produced broadly consistent findings. Among 62 adults with overweight, a low-fat vegan diet reduced body weight and improved HOMA-IR and oral glucose insulin sensitivity, whereas these measures did not change significantly during a Mediterranean-diet phase [41]. Nevertheless, energy intake and weight decreased more during the vegan phase, again limiting attribution to the exclusion of animal-derived foods.

#### 3.2.3. Dietary Composition, β-Cell Function, and Evidence Limitations

The composition of healthful PBDs influences insulin sensitivity through pathways beyond weight loss. They are generally lower in saturated fat and higher in unsaturated fat, a composition which favourably alters lipid partitioning. In a human overfeeding trial, saturated fat increased liver fat and circulating ceramides more than polyunsaturated fat, providing indirect support for a role of dietary fat quality in ectopic lipid-mediated IR [69]. Fibre-rich, minimally processed plant foods additionally reduce postprandial glycaemic exposure by slowing carbohydrate absorption. However, these component effects should not be interpreted as evidence that animal-food exclusion itself improves insulin signaling.

PBDs may also improve pancreatic β-cell responsiveness. In a 16-week randomized trial of 75 adults with overweight and no history of diabetes, low-fat PBDs increased meal-stimulated insulin secretion and β-cell glucose sensitivity while reducing HOMA-IR compared with no dietary change [70]. These findings suggest improved coordination between circulating glucose concentrations and insulin secretion rather than simply increased insulin output. β-cell function was estimated using mathematical modelling of meal-test data rather than direct measures of β-cell mass or intracellular signaling; the findings therefore indicate improved functional responsiveness rather than structural restoration.

Evidence from more tightly controlled diets further indicates that plant origin alone may not determine the response. In a small five-week trial using hyperinsulinaemic-euglycaemic clamps, energy- and protein-matched high-protein vegan and omnivorous diets improved peripheral insulin sensitivity to a similar extent in adults with T2D, with no significant improvement in hepatic insulin sensitivity in either group [71]. Although the vegan diet was mycoprotein-rich and differed from minimally processed PBDs, the findings emphasize the importance of energy balance, macronutrient composition, and food quality.

Overall, human evidence supports improved insulin sensitivity during low-fat, minimally processed PBD interventions, particularly when accompanied by reductions in body weight, visceral adiposity, and ectopic lipid. Whether these diets exert effects independent of energy intake and weight loss remains uncertain. Future trials should use energy- and weight-matched comparators, hyperinsulinaemic-euglycaemic clamps, tissue imaging, and molecular analyses to distinguish changes in hepatic, skeletal-muscle, and adipose-tissue insulin action.

### 3.3. Gut Microbiota and the Gut–Brain Axis

Beyond their effects on food structure, energy intake, and insulin sensitivity, PBDs influence metabolic regulation through the gut microbiota, which converts indigestible dietary components into bioactive metabolites. Relevant functions include fermentation of plant polysaccharides, production of SCFAs, modification of bile acids, and interaction with enteroendocrine, immune, and neural pathways. Human evidence, however, is stronger for changes in microbial function and metabolite signaling than for consistent changes in individual bacterial taxa.

#### 3.3.1. Microbiota Composition and Function

Habitual vegan, vegetarian, and omnivorous diets are associated with distinguishable metagenomic profiles. In a multinational study of 21,561 individuals, vegan-associated microbial signatures correlated with more favourable cardiometabolic markers, whereas several microbes associated with red-meat consumption correlated with less favourable profiles [72]. Similar microbial features were also observed in omnivores consuming more healthy plant foods, suggesting that plant-food quality and diversity may be more important than dietary labels alone. Because these findings were cross-sectional, they cannot establish whether microbial differences contributed to the metabolic outcomes.

Interventional evidence remains heterogeneous. A systematic review of 12 human studies found potentially favourable effects of PBDs on microbial composition, although results for several commonly reported taxa, including *Faecalibacterium* and *Coprococcus*, were inconsistent [73]. In adults with overweight, a 16-week low-fat vegan intervention increased the relative abundance of *Faecalibacterium prausnitzii*, and changes in this species were weakly associated with reductions in body weight, fat mass, and visceral adiposity. Changes in *Bacteroides fragilis* were also related to weight and insulin-sensitivity outcomes [74]. These associations do not demonstrate that microbiota changes mediated the effects of the diet.

Microbial function is therefore more informative than taxonomic composition in this context. In a 17-week intervention, increasing dietary fibre raised the abundance of microbial carbohydrate-degrading enzymes without increasing microbial diversity. Immune responses also differed according to baseline microbiota characteristics [75]. Thus, greater fibre intake does not necessarily produce a uniform increase in diversity or inflammatory improvement.

#### 3.3.2. Short-Chain Fatty Acids and Gut–Brain Signaling

Acetate, propionate, and butyrate are generated through microbial fermentation of fermentable fibres and resistant starch. SCFAs activate FFAR2 and FFAR3 on intestinal enteroendocrine cells, promoting secretion of PYY and GLP-1 [76,77,78]. They also influence energy metabolism and neural pathways, although receptor-specific causality has been established more clearly in experimental models than in humans.

In individuals with T2D, a diet enriched with diverse fibres selectively promoted a limited group of SCFA-producing bacterial strains. Greater enrichment of these strains was associated with better glycaemic control and other metabolic improvements, accompanied by changes in GLP-1 secretion [79]. This supports the fibre–microbiome component of the proposed mechanism but does not directly establish the effects of complete PBDs.

More direct evidence comes from SCFA-administration studies. In males with overweight or obesity, colonic infusion of physiologically relevant SCFA mixtures increased fat oxidation, energy expenditure, and circulating PYY while reducing lipolysis [76]. Targeted colonic propionate delivery has similarly increased PYY and GLP-1, reduced subsequent energy intake, and limited weight gain and abdominal fat accumulation [44]. These studies demonstrate that microbial metabolites can affect human metabolic and enteroendocrine regulation, although experimentally delivered SCFAs are not equivalent to consuming PBDs.

Human neuroimaging provides a more direct connection with the gut–brain axis. In a randomized crossover study, colonic propionate delivery reduced activation of the caudate and nucleus accumbens in response to high-energy food images. This was accompanied by lower food appeal and reduced ad libitum energy intake but was not explained by changes in circulating PYY or GLP-1 [80]. Propionate may therefore influence reward-related eating through striatal pathways. Nevertheless, the participants were healthy males without obesity, and the intervention did not test a whole dietary pattern.

#### 3.3.3. Energy Harvest and Evidence Limitations

The gut microbiota has also been proposed to influence obesity through differences in energy extraction from food. However, the historically proposed Firmicutes-to-Bacteroidetes ratio has shown substantial inconsistency across human studies and cannot be considered a reliable taxonomic marker of obesity [81]. A systematic review of food-based weight-loss interventions found minimal overall changes in microbial diversity and faecal SCFAs, with inconsistent changes in individual taxa [82].

Microbial metabolites may also support intestinal barrier integrity and reduce translocation of pro-inflammatory products such as lipopolysaccharide. However, few human dietary trials have measured microbial function, intestinal permeability, circulating endotoxin, and adiposity together [83]. These pathways should therefore be regarded as plausible rather than established mediators. Overall, human evidence supports a diet-responsive microbiome and metabolically active microbial products, but direct proof that microbiota changes mediate PBD-induced weight loss remains limited.

### 3.4. Adipogenesis and Adipose Tissue Biology

Adipose tissue is now recognized as a metabolically active endocrine organ rather than a passive reservoir for energy storage [84]. It plays a central role in regulating whole-body energy homeostasis through lipid storage, adipokine secretion, immune modulation, and endocrine communication with peripheral organs, including the liver, skeletal muscle, pancreas, and brain [84,85]. Healthy expansion of adipose tissue depends on adipogenesis, the tightly regulated differentiation of preadipocytes into mature adipocytes. This process enables excess lipids to be distributed across newly formed adipocytes rather than accumulating within existing cells, thereby preserving insulin sensitivity and maintaining normal adipose tissue function [85,86]. In obesity, the capacity for adipogenesis becomes impaired, and adipose tissue expansion shifts toward adipocyte hypertrophy rather than hyperplasia [87]. Hypertrophic adipocytes exhibit reduced lipid storage capacity, impaired insulin signaling, mitochondrial dysfunction, and increased susceptibility to cellular stress [86]. As adipocytes enlarge, oxygen diffusion becomes insufficient, resulting in local hypoxia, extracellular matrix remodelling, fibrosis, and chronic activation of inflammatory pathways [84,86]. These structural and metabolic alterations contribute to adipose tissue dysfunction and promote systemic IR, a hallmark of obesity-associated metabolic disease.

White adipose tissue (WAT) is the predominant adipose tissue depot in adults and serves as the principal site for triglyceride storage and endocrine regulation [88]. Beyond lipid storage, WAT functions as an endocrine organ by secreting adipokines and inflammatory mediators, including leptin, adiponectin, resistin, tumour necrosis factor-α (TNF-α), and interleukin-6 (IL-6), which regulate appetite, glucose metabolism, inflammation, and energy homeostasis [84,85,89]. In healthy individuals, adipokine secretion maintains metabolic homeostasis. However, obesity profoundly alters this secretory profile, resulting in increased production of pro-inflammatory cytokines [88]. Concurrently, obesity promotes infiltration of pro-inflammatory M1 macrophages into adipose tissue, amplifying chronic low-grade inflammation through activation of Nuclear Factor Kappa B (NF-κB) signaling and other inflammatory pathways [90]. This persistent inflammatory state contributes to impaired insulin signaling and accelerates the development of metabolic syndrome and T2D [85,86].

Not all adipose tissue depots exhibit the same metabolic properties. Visceral adipose tissue (VAT), located within the abdominal cavity surrounding internal organs, is metabolically distinct from subcutaneous adipose tissue (SAT) [91]. Compared with SAT, VAT demonstrates greater lipolytic activity and releases larger quantities of free fatty acids directly into the portal circulation, exposing the liver to an increased lipid load [67,92]. Elevated portal free fatty acid delivery promotes hepatic steatosis, increased gluconeogenesis, hepatic IR, and dyslipidemia [67,86]. Furthermore, VAT exhibits greater inflammatory activity and altered adipokine secretion, contributing to systemic metabolic dysfunction and thereby increasing the risk of cardiometabolic disease [93]. Consequently, visceral adiposity is considered a stronger predictor of IR and obesity-related complications than total body fat alone.

Beyond total energy intake, dietary fat quality also influences adipose tissue distribution and metabolic function. In a randomized controlled overfeeding trial, diets enriched in saturated fatty acids resulted in significantly greater accumulation of visceral and hepatic fat, whereas polyunsaturated fatty acids preferentially promoted lean tissue accretion and were associated with substantially lower ectopic fat deposition despite similar caloric excess [94]. These findings highlight that dietary fat quality, rather than energy intake alone, plays an important role in regulating adipose tissue distribution and metabolic health.

Brown adipose tissue (BAT) and inducible beige adipocytes differ from WAT in that they dissipate energy through Uncoupling Protein-1 (UCP1)-mediated thermogenesis [95,96]. Human imaging confirms metabolically active BAT in adults, with BAT activity generally associated with younger age and lower adiposity [97,98]. As these tissues primarily concern energy expenditure rather than adipogenesis, detailed evidence on BAT activation, beige adipocyte recruitment, and plant-derived compounds such as capsinoids is discussed in the Energy Expenditure and Thermogenesis section.

Direct human evidence that PBDs modify adipogenesis or induce adipose browning remains limited. In a 16-week randomized trial of 244 adults, a low-fat vegan diet reduced visceral adipose tissue volume by 216.5 cm^3^ versus 7.7 cm^3^ with no dietary change (between-group difference, −208.8 cm^3^; *p* < 0.001), alongside improvements in insulin sensitivity [29]. However, these outcomes do not establish direct effects on adipocyte differentiation or browning. Current human evidence therefore supports effects of PBD interventions on adipose tissue quantity and distribution more strongly than on adipogenesis itself.

### 3.5. Chronic Low-Grade Inflammation

Chronic low-grade inflammation is a central feature of obesity-related metabolic dysfunction. Expansion and remodelling of adipose tissue promote immune-cell recruitment and activation of inflammatory pathways, including NF-κB and the NOD-like receptor family pyrin domain containing 3 (NLRP3) inflammasome, thereby contributing to impaired insulin signaling and systemic metabolic dysfunction [99,100,101]. PBDs may attenuate this inflammatory state through reductions in adiposity, improvements in dietary fat quality and gut barrier function, and greater intake of fibre and plant-derived bioactive compounds. However, direct mechanistic evidence from whole-diet human studies remains limited.

#### 3.5.1. Adipose Tissue Inflammation and Macrophage Recruitment

In obesity, adipocyte hypertrophy, local hypoxia, extracellular-matrix remodelling, and cellular stress promote recruitment of circulating monocytes and accumulation of adipose-tissue macrophages [99,100]. Compared with macrophages in metabolically healthy adipose tissue, those accumulating during obesity more frequently exhibit pro-inflammatory phenotypes and secrete mediators including TNF-α, interleukin-1 beta (IL-1β), and IL-6, while anti-inflammatory signaling is relatively reduced [99,100]. This shift amplifies local inflammation and interferes with insulin action through stress-response pathways including NF-κB and c-Jun N-terminal kinase [99].

Human studies support the metabolic relevance of adipose-tissue macrophage accumulation. In adults with metabolic syndrome, greater macrophage infiltration and the presence of crown-like structures in SAT were associated with higher IR and circulating inflammatory markers [102]. Other human studies have similarly shown that adipose-tissue macrophage abundance, assessed using markers including CD68, CD14, and CD206, increases with adiposity and is associated with impaired insulin sensitivity [103]. These findings support macrophage activation as an important link between dysfunctional adipose-tissue expansion and systemic metabolic disturbance.

Obesity is also associated with higher circulating concentrations of inflammatory biomarkers, including C-reactive protein (CRP), TNF-α, IL-6, and IL-1β [99]. Importantly, this inflammatory phenotype is at least partly reversible. In a randomized lifestyle intervention, approximately 10% weight loss was accompanied by reductions in CRP and IL-6, improved insulin sensitivity, and lower markers of vascular inflammation [104]. These findings indicate that obesity-associated inflammation is closely linked to adiposity and energy balance, which complicates attempts to identify diet-specific anti-inflammatory effects.

#### 3.5.2. NF-κB and NLRP3 Inflammasome Signaling

NF-κB is a major transcriptional regulator linking metabolic stress to inflammatory gene expression in obesity. Free fatty acids, oxidative stress, cytokines, and other signals associated with adipose-tissue dysfunction can activate NF-κB, increasing expression of inflammatory mediators including TNF-α, IL-6, and monocyte chemoattractant protein-1. Persistent NF-κB activation in turn impairs insulin-receptor signaling and propagates inflammation across adipose tissue, liver, and skeletal muscle [105].

Human intervention studies indicate that this pathway is modifiable. In the GENOBIN study, dietary weight loss in adults with metabolic syndrome was associated with reduced expression of several genes involved in NF-κB signaling in peripheral blood mononuclear cells, alongside improvements in insulin sensitivity [106]. Similarly, supplementation with the polyphenols epigallocatechin gallate and resveratrol altered adipose-tissue gene-expression profiles related to inflammation, oxidative stress, and immune activation in adults with overweight or obesity [107]. These studies support biological responsiveness of NF-κB-related pathways, although neither establishes a specific effect of complete PBDs.

The NLRP3 inflammasome provides a second link between metabolic stress and inflammation. Lipotoxicity, mitochondrial dysfunction, oxidative stress, and nutrient excess can activate NLRP3, promoting caspase-1-dependent maturation of IL-1β and IL-18 [108]. These cytokines can further impair insulin signaling and β-cell function. Human adipose-tissue studies have reported increased expression of NLRP3, caspase-1, and IL-1β in obesity, with greater activation associated with IR [109]. Weight loss following bariatric surgery has also been associated with reduced inflammasome-related gene expression and attenuation of adipose inflammation, suggesting that NLRP3 activity is at least partly reversible [110].

#### 3.5.3. Plant-Based Dietary Modulation of Inflammation

PBDs influence inflammatory pathways through several interacting mechanisms. Higher intakes of fibre and plant-derived bioactive compounds affect gut microbial metabolism, intestinal barrier integrity, oxidative stress, and inflammatory signaling. Reduced translocation of bacterial lipopolysaccharide decreases toll-like receptor 4 (TLR4)-NF-κB activation, while fibre-derived SCFAs support epithelial barrier integrity and immune regulation [111]. However, few human PBD trials have simultaneously assessed intestinal permeability, adipose-tissue inflammation, and metabolic outcomes, so these pathways remain biologically plausible rather than established [83].

Human dietary studies more consistently demonstrate changes in circulating inflammatory markers than direct effects on tissue-level inflammatory signaling. A 16-week low-fat vegan intervention reduced body weight, visceral adiposity, and IR in adults with overweight [112], changes that would be expected to lessen inflammatory burden, although the independent contribution of dietary composition cannot be separated from concurrent weight loss. Similarly, the PREDIMED trial showed that a Mediterranean dietary pattern enriched with extra-virgin olive oil or nuts reduced several circulating markers of vascular inflammation [113]. Although this supports the anti-inflammatory potential of PBDs, it is important to note that a Mediterranean diet is not equivalent to a vegan or vegetarian diet.

Overall, human evidence supports an association between PBDs, lower adiposity, and a more favourable inflammatory profile. However, direct evidence that whole PBDs suppress NF-κB or NLRP3 signaling independently of weight loss remains limited. Future mechanistic trials should integrate circulating inflammatory biomarkers with adipose-tissue, immune-cell, and gut-barrier measurements to determine whether inflammatory modulation is a primary effect of PBDs or largely secondary to reductions in energy intake and adiposity.

### 3.6. Oxidative Stress

Oxidative stress is an important component of obesity-associated metabolic dysfunction and arises when the production of reactive oxygen species (ROS) exceeds endogenous antioxidant capacity. Chronic nutrient excess, adipocyte hypertrophy, and elevated lipid flux increase metabolic stress in adipose tissue and other insulin-sensitive organs, promoting mitochondrial dysfunction, impaired oxidative phosphorylation, and accumulation of ROS [114,115]. These disturbances can interfere with insulin signaling, promote lipid accumulation, and reinforce chronic inflammatory pathways.

Endoplasmic reticulum stress provides an additional link between nutrient excess and metabolic dysfunction. Increased demands on protein folding and lipid handling activate the unfolded protein response, which may become chronically engaged in obesity [114,116]. Cellular defence against oxidative stress is coordinated in part by nuclear factor erythroid 2-related factor 2 (Nrf2), a transcription factor that promotes expression of antioxidant and detoxification enzymes, including heme oxygenase-1, NADPH quinone dehydrogenase 1, and glutathione-related enzymes [117,118,119]. Nrf2-related pathways represent a potential mechanism relevant to dietary modulation of oxidative stress; however, direct demonstration of this effect after whole PBD interventions in humans remains limited [117].

Oxidative stress also overlaps with nitrosative and carbonyl stress. Increased production of reactive nitrogen species and formation of peroxynitrite can damage proteins, lipids, and nucleic acids and impair metabolic signaling [120,121,122]. Reactive carbonyl species generated during lipid peroxidation and glucose metabolism similarly promote formation of advanced glycation end products (AGEs), which interact with their receptor (RAGE) to amplify ROS production and inflammatory signaling [123]. These pathways are biologically relevant to obesity-associated IR, but most mechanistic evidence does not derive from whole-diet PBD trials.

PBDs improve redox balance through several complementary mechanisms, including greater intake of antioxidant-rich foods, lower saturated-fat exposure, and changes in postprandial metabolism. Human intervention evidence is strongest for changes in circulating oxidative and inflammatory biomarkers rather than direct measurements of intracellular redox signaling. In a randomized controlled trial of adults with overweight or obesity, increased fruit and vegetable consumption reduced circulating lipopolysaccharide-binding protein, a marker associated with metabolic endotoxemia and low-grade inflammation [124]. A 12-week trial provided plant-based meals on five days per week and self-selected meals on two days; both groups followed the same calorie limits of 1300 kcal/day for females and 1600 kcal/day for males. Weight loss was 6.56 versus 5.11 kg with conventional calorie restriction in the per-protocol analysis (both *p* < 0.05 versus baseline), with no significant between-group difference (−1.44 kg; 95% CI, −3.27 to 0.39); non-inferiority was demonstrated in the per-protocol analysis (*p* = 0.019) but was not confirmed by the intention-to-treat confidence interval. Improvements in insulin sensitivity, estimated visceral fat mass, and inflammatory markers (IL-8 and TNF-α) were significant within the plant-based group (all *p* < 0.05), whereas corresponding changes within the control group were not significant; between-group differences for these outcomes were also not significant [125].

More direct evidence comes from a randomized crossover trial comparing a vegan tofu-based meal with an energy- and macronutrient-matched conventional meal in males with diabetes or obesity. The plant-based meal reduced postprandial oxidative and dicarbonyl stress, increased reduced glutathione concentrations, and lowered methylglyoxal, a reactive dicarbonyl species involved in carbonyl stress [126]. As this study assessed a single meal rather than a sustained whole-diet intervention, it provides evidence supporting acute metabolic responsiveness but does not establish long-term redox effects of PBDs.

Overall, current human evidence suggests that plant-rich diets and PBDs may reduce oxidative burden, particularly in the postprandial state and alongside improvements in adiposity and insulin sensitivity. However, direct evidence that whole PBDs modify intracellular ROS production, Nrf2 signaling, or AGE-RAGE activity independently of weight loss remains limited. Future mechanistic trials should combine whole-diet interventions with tissue-level measures of redox status, mitochondrial function, antioxidant signaling, and inflammatory pathways to clarify whether improved oxidative balance is a primary dietary effect or a secondary consequence of reduced energy intake and adiposity.

### 3.7. Lipid Metabolism

Obesity is characterized not only by expanded adipose mass but by a distinctive derangement of lipid handling: elevated low-density lipoprotein (LDL) cholesterol (one of the major risk factors of cardiovascular morbidity and mortality), raised triglycerides, and the deposition of lipid in tissues not designed to store it [10]. PBDs act on each of these through mechanisms characterized in human interventional studies, operating at the level of intestinal sterol absorption, hepatic receptor expression, and intracellular lipid partitioning.

#### 3.7.1. Sterol Absorption and Hepatic LDL Receptor Upregulation

The best-defined mechanism concerns enterohepatic cholesterol handling [127]. Viscous fibres, abundant in oats, barley, legumes, and psyllium, form a gel matrix within the intestinal lumen that thickens the unstirred water layer and interferes with micelle formation, which traps bile acids and thereby limits their reabsorption at the terminal ileum [128]. Reduced reabsorption lowers hepatocyte cholesterol and bile acid content, which triggers upregulation of hepatic LDL receptors that clear apolipoprotein B-containing lipoproteins, including LDL particles, from the circulation [128]. The pathway is mechanistically identical to that exploited by pharmacological bile acid sequestrants, and it explains why the cholesterol-lowering effect is largely confined to soluble rather than insoluble fibre [129]. A parallel limb operates through postprandial glycemia: lower glucose excursions reduce insulin secretion, and since insulin activates HMG-CoA reductase, the rate-limiting enzyme of cholesterol biosynthesis, the reduction in insulin translates into diminished endogenous cholesterol synthesis [130].

Plant sterols and stanols, structurally analogous to cholesterol and present throughout plant foods, contribute an additional absorptive block by competing for incorporation into mixed micelles. In a randomized, placebo-controlled crossover trial in 32 adults with primary hypercholesterolemia, supplementation with 1.8 g/day of free plant sterols and stanols over six weeks reduced LDL-cholesterol by 4.9% and total cholesterol by 2.8% relative to control, with no significant change in HDL-cholesterol or triglycerides [131]. The mechanism converges on the same hepatic endpoint: reduced intestinal cholesterol absorption lowers hepatic cholesterol content, upregulating LDL receptor expression and enhancing clearance of LDL and other apolipoprotein B-containing particles [131].

#### 3.7.2. Food Matrix Effects Beyond Nutrient Substitution

These mechanisms operate through the plant food matrix rather than merely through the avoidance of saturated fat as demonstrated by isocaloric feeding studies holding fat composition constant. In a randomized trial of 120 adults with LDL cholesterol between 3.3 and 4.8 mmol/L, two diets matched for total fat, saturated fat, protein, carbohydrate, and cholesterol were compared over four weeks at constant body weight; the arm incorporating substantially more vegetables, legumes, and whole grains achieved additional reductions of 0.22 mmol/L in total cholesterol and 0.18 mmol/L in LDL-cholesterol relative to the conventional low-fat arm [132]. The authors concluded that guidelines emphasizing avoidance of saturated fat and cholesterol alone had underestimated the LDL-lowering potential achievable by actively including nutrient-dense plant foods [132]. As weight and macronutrient profile were held constant, the incremental effect is attributable to the plant matrix itself, principally its viscous fibre and phytosterol content.

#### 3.7.3. Ectopic Lipid Deposition

Ectopic lipid deposition in the liver and skeletal muscle provides an important link between disturbed lipid partitioning and IR. In a 16-week low-fat vegan randomized clinical trial, magnetic resonance spectroscopy demonstrated reductions in hepatocellular and intramyocellular lipid alongside improved insulin sensitivity [29]. These findings support redistribution of intracellular lipid as a potential mechanism, although concurrent reductions in energy intake and body weight preclude attribution to dietary composition alone. The possible relationship between ectopic lipid depletion and postprandial energy expenditure is considered in the following section.

### 3.8. Energy Expenditure and Thermogenesis

Weight loss on PBDs is frequently attributed solely to reduced energy intake, but human interventional data suggest that increased postprandial energy expenditure occurs concurrently. The thermic effect of food, the energy cost of digesting, absorbing, and storing ingested nutrients, accounts for approximately 10% of total energy expenditure and is measurable in humans by indirect calorimetry [133].

#### 3.8.1. Postprandial Energy Expenditure

The clearest evidence comes from a 16-week randomized clinical trial in 244 adults with a BMI of 28 to 40 kg/m^2^, in which participants were assigned either to a low-fat vegan diet (approximately 75% of energy from carbohydrate, 15% protein, and 10% fat, comprising vegetables, grains, legumes, and fruits without animal products or added fats) or to no dietary change [29]. The thermic effect of food was measured by indirect calorimetry over three hours following a standardized 720 kcal liquid breakfast. The intervention increased the thermic effect of food, with a 14.1% between-group difference in effect size (95% CI, 6.5–20.4%; *p* < 0.001). Body weight decreased by 6.4 kg in the intervention group compared with 0.5 kg in the control group, corresponding to a between-group treatment effect of −5.9 kg [29]. Critically, the design included an untreated control group, which resolved a limitation of earlier work in which the comparator followed an active National Cholesterol Education Program diet, leaving the thermogenic effect of the vegan pattern indeterminate [29]. These findings support increased postprandial thermogenesis as a biologically plausible contributor to weight loss.

#### 3.8.2. Mechanistic Basis

The thermogenic response appears coupled to the changes in lipid partitioning described above rather than independent of them. When both study groups were analysed together, changes in the thermic effect of food correlated negatively with changes in fat mass (r = −0.30; *p* < 0.05) and positively with changes in predicted insulin sensitivity (PREDIM; r = 0.36; *p* < 0.05) [29]. However, these associations were not significant within the intervention group alone, and therefore do not establish that improvements in insulin sensitivity or fat mass directly mediated the increase in postprandial thermogenesis. These concurrent changes are consistent with a possible relationship between improved lipid partitioning, insulin sensitivity, and postprandial energy metabolism, although the trial did not directly assess mitochondrial function or establish a causal pathway linking ectopic lipid reduction to increased thermogenesis [29]. Human feeding studies provide indirect mechanistic support, as short-term high-fat dietary exposure has been shown to reduce skeletal-muscle mitochondrial respiratory capacity under experimental conditions [134]. Macronutrient composition also influences thermogenesis, with reported diet-induced thermogenesis values of approximately 20–30% of ingested nutrient energy for protein, 5–10% for carbohydrate, and 0–3% for fat [133].

#### 3.8.3. Brown and Beige Adipose Tissue

A further thermogenic mechanism, activation of brown and beige adipose tissue, has attracted considerable interest, and several plant-derived compounds demonstrably engage it in humans. Capsinoids, non-pungent capsaicin analogues from chilli peppers, increase whole-body energy expenditure in a manner dependent on BAT status, as confirmed by 18F-fluorodeoxyglucose positron emission tomography, and six weeks of capsinoid treatment raised supraclavicular BAT activity by 48.8% [135,136]. Catechin-rich green tea extract has similarly shown reproducible thermogenic effects in humans, with a synergistic contribution from caffeine [137]. These findings should nonetheless be interpreted with restraint in the context of dietary patterns. The evidence pertains to isolated compounds delivered at supplemental doses rather than to habitual plant-based eating; no trial has tested whether PBDs activate BAT in humans, and much of the underlying browning literature derives from rodent and cell-culture models awaiting human validation [137]. That human BAT is not readily recruited is underscored by a randomized controlled trial finding no evidence of activation after 24 weeks of supervised exercise training in young sedentary adults [138]. Brown and beige thermogenesis therefore remains a biologically plausible but currently unproven contributor to the effects of PBDs on energy expenditure.

### 3.9. Epigenetic Regulation

Epigenetic mechanisms, principally deoxyribonucleic acid (DNA) methylation, histone modification, and non-coding ribonucleic acid (RNA) regulation, allow gene expression to be adjusted in response to nutritional stimuli without alteration of the underlying sequence [139]. In the context of obesity, these processes are of interest for two distinct reasons: they provide a mechanism through which early-life nutritional exposure programmes lifelong adiposity risk, and they represent a substrate on which dietary intervention in adulthood acts [140]. Human evidence now exists for both.

#### 3.9.1. Developmental Programming and Maternal Nutrition

The most direct demonstration that periconceptional nutrition leaves durable epigenetic marks comes from the Dutch Hunger Winter of 1944 to 1945. Individuals exposed to famine in utero were found six decades later to carry lower DNA methylation of the imprinted insulin-like growth factor 2 (IGF2) differentially methylated region than their unexposed same-sex siblings, an association specific to periconceptional exposure and consistent with a deficiency in methyl donors such as methionine [141]. The finding provided the first empirical support in humans for the proposition that early environmental conditions produce epigenetic changes persisting across the lifespan [141]. Subsequent in-depth characterization across five differentially methylated regions of the IGF2/H19 locus in 60 exposed individuals and 60 sibling controls confirmed small but consistent differences at all IGF2 regions after adjustment for multiple testing [142]. Genome-scale analysis extended these observations beyond a single locus, demonstrating that prenatal malnutrition-associated differentially methylated regions occur preferentially at regulatory regions, map to genes enriched for differential expression during early development, and extend along pathways related to growth and metabolism; regions located in INSR and CPT1A showed enhancer activity in vitro, with differential methylation associated with birth weight and serum LDL-cholesterol [143]. Prenatal famine exposure is associated with higher BMI in later life, suboptimal glucose handling, and elevated total and LDL-cholesterol [143]. Comparable methylation differences have been reported following other adverse prenatal exposures, including gestational diabetes and maternal smoking, and maternal glycemia has been associated with altered placental leptin gene methylation, a plausible route to long-term programming of excess adiposity [144]. Maternal nutrition therefore establishes an epigenetic substrate for offspring adiposity, positioning periconceptional dietary quality as a determinant of intergenerational metabolic health.

#### 3.9.2. Dietary Modification of the Adult Methylome

That epigenetic marks remain modifiable in adulthood has been demonstrated by the 18-month DIRECT PLUS randomized controlled trial, which compared healthy dietary guidelines, a Mediterranean diet, and a polyphenol-rich, low red and processed meat green-Mediterranean diet in participants with abdominal obesity or dyslipidemia [39]. Among 260 participants with a mean baseline BMI of 31.2 kg/m^2^, the green-Mediterranean intervention was associated with substantially more intervention-specific differentially methylated regions (DMRs) than the conventional Mediterranean and healthy dietary-guideline interventions, with 1573 DMRs identified after 18 months in the green-Mediterranean arm [39]. Transcriptomic analysis likewise identified 1753 differentially expressed genes (DEGs) in this arm, indicating broader transcriptional changes alongside the methylation response [39]. Changes in folate and vitamin B12, both involved in one-carbon metabolism, were highlighted as potential contributors to the observed epigenetic response, while network analysis identified candidate genes associated with changes in serum folic acid that linked transcriptional and phenotypic responses [39]. The magnitude of the contrast between the two Mediterranean arms is informative: both consumed walnuts and differed principally in polyphenol load, with the green arm additionally receiving green tea and Mankai (a cultivated aquatic plant, Wolffia globosa or duckweed, rich in polyphenols and protein), with the green-Mediterranean arm showing the largest epigenetic response [39]. Polyphenols are thought to act in part through inhibition of DNA methyltransferase 1, the enzyme catalyzing maintenance methylation [145].

#### 3.9.3. Epigenetic Aging

Methylation-based estimates of biological age provide a further readout. In the CENTRAL trial, which compared low-fat and Mediterranean/low-carbohydrate diets, attenuation of methylation age was significantly greater among participants achieving successful weight loss and improved MRI-assessed liver fat status over 18 months [146]. Evidence at the level of dietary pattern is nonetheless mixed: one-year adherence to a Mediterranean diet in the NU-AGE study was associated with lower biological age acceleration by the Horvath clock, whereas the CENTRAL nutrition intervention showed no effect on epigenetic methylation age by a 240-CpG algorithm [147]. The systematic review from which these comparisons are drawn concluded that healthy dietary patterns appear to exert positive effects on DNA methylation, while noting that larger randomized trials remain necessary to confirm this [147].

### 3.10. Clinical Consequences of Obesity

**Figure 2:**
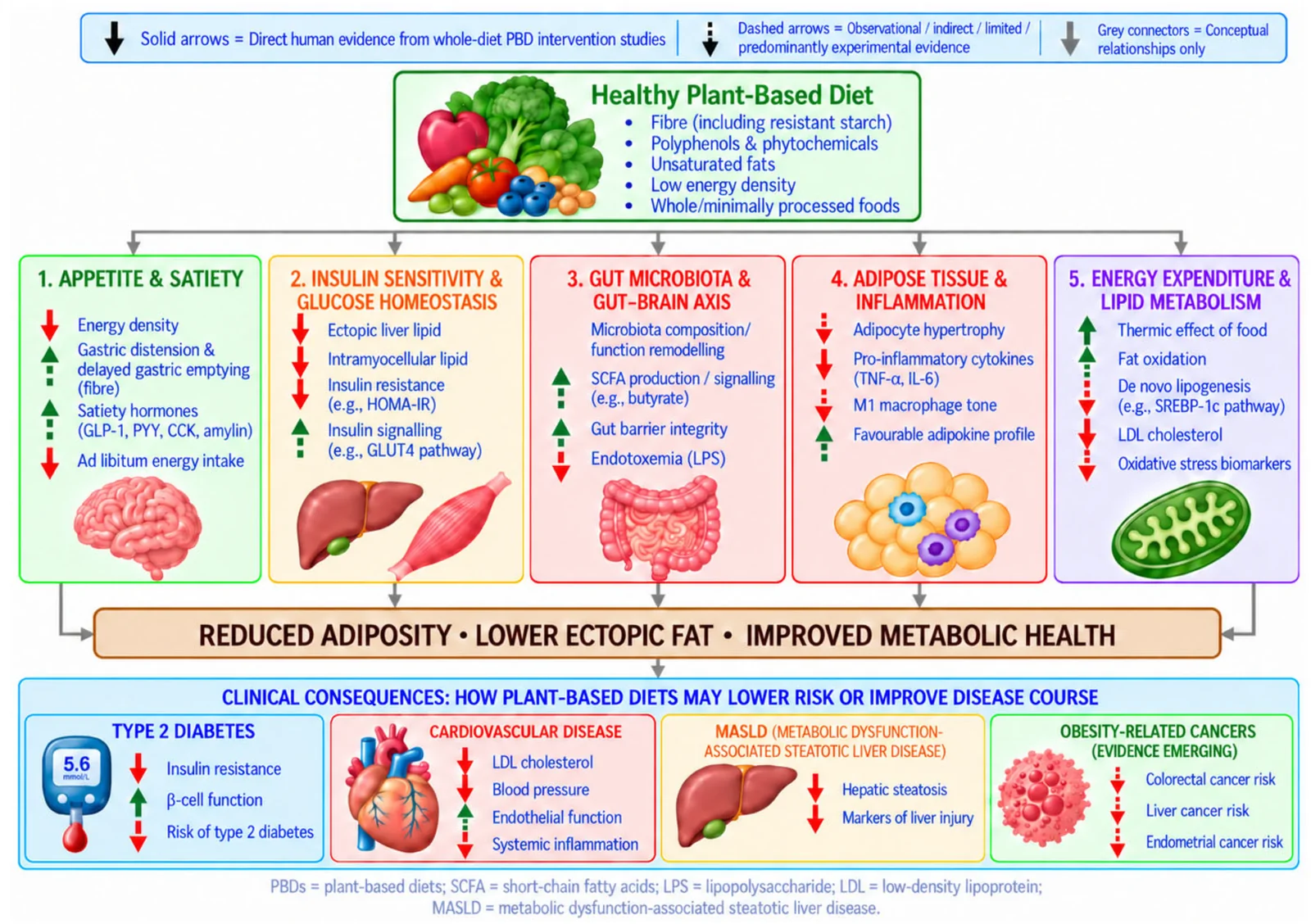
Cellular and molecular mechanisms linking healthy plant-based diets to reduced adiposity and obesity-related clinical outcomes.

The cellular and molecular disturbances described above converge over time to produce the major chronic diseases associated with obesity. As summarized in Figure 2, IR, chronic low-grade inflammation, ectopic lipid accumulation, oxidative stress, dysregulated adipokine signaling, and impaired energy homeostasis form an interconnected mechanistic network linking excess adiposity with T2D, CVD, MASLD, and selected obesity-related cancers. Healthy PBDs influence these outcomes by acting simultaneously on several of these upstream pathways, although the strength and directness of the human evidence vary by clinical endpoint.

Healthy plant-based diets (PBDs) may influence obesity through interconnected effects on appetite and satiety, insulin sensitivity and glucose homeostasis, the gut microbiota–gut–brain axis, adipose tissue and inflammation, and energy expenditure and lipid metabolism. Together, these pathways may contribute to reduced adiposity, lower ectopic fat, and improved metabolic health, thereby potentially lowering the risk of, or improving the course of, type 2 diabetes, cardiovascular disease, and metabolic dysfunction-associated steatotic liver disease (MASLD). Evidence linking PBDs with lower risk of selected obesity-related cancers is emerging. Solid arrows indicate effects supported by direct human whole-diet PBD intervention studies, whereas dashed arrows indicate observational associations or indirect, limited, or predominantly experimental evidence. Green arrows indicate increases and red arrows indicate decreases in the corresponding biological or clinical parameters. Grey connectors represent conceptual relationships rather than established causal mediation. The depicted pathways do not establish that PBDs prevent disease through these mechanisms or that every PBD improves every endpoint. Created using BioRender and ChatGPT.

#### 3.10.1. Cancer

Excess adiposity is an established cause of at least 13 cancer types, including adenocarcinoma of the esophagus, gastric cardia, colon and rectum, liver, gallbladder, pancreas, postmenopausal breast, endometrium, ovary, kidney, and thyroid, as well as meningioma and multiple myeloma [148,149]. Consensus panels have concluded that these cancers have sufficient evidence and biological plausibility linking excess body fatness as a cause, and collectively they account for a substantial share of the global cancer burden [148]. The mechanisms overlap closely with those described throughout this review: hyperinsulinemia and elevated insulin-like growth factor signaling, chronic low-grade inflammation, adipokine imbalance, and for hormone-sensitive sites, increased peripheral aromatization and estrogen exposure [148,149]. By acting on these same upstream pathways, healthful PBDs plausibly attenuate the risk of several obesity-related cancers, with the most consistent human evidence for colorectal, endometrial, and liver cancer.

For colorectal cancer, a meta-analysis of prospective cohorts incorporating more than 1.2 million participants demonstrated a significant inverse association between PBDs and cancer incidence, an association that strengthened when the pattern specifically emphasized healthful plant foods such as fruits, vegetables, whole grains, legumes, and nuts [150]. Mechanistically, these effects partly reflect modulation of the gut microbiota and increased production of SCFAs, particularly butyrate, which inhibit tumour cell proliferation, promote apoptosis, and reduce systemic inflammation and oxidative DNA damage [151].

For liver cancer, plant-based indices show a similarly favourable and comparatively large effect, with a large prospective cohort study reporting lower risk per increment in the healthful PBD index [152]. Endometrial cancer, the malignancy most strongly linked to obesity, is biologically responsive to the same insulin-resistance and estrogen-mediated pathways that PBDs favourably modify; across site-specific analyses, higher adherence to overall and healthful PBDs has been associated with lower risk at the cancer sites most consistently studied, whereas unhealthful PBDs have been associated with higher risk [153]. These associations are strongest for whole, healthful PBDs and are attenuated or reversed when PBDs are dominated by refined and processed foods, reinforcing that diet quality, not merely plant origin, governs the effect.

#### 3.10.2. Type 2 Diabetes Mellitus

Obesity increases the risk of T2D through the combined effects of IR, ectopic lipid accumulation, chronic inflammation, and progressive β-cell dysfunction. Visceral adiposity and excess fatty-acid flux impair insulin action in skeletal muscle and liver, while lipotoxicity, oxidative stress, and inflammatory signaling place increasing metabolic stress on pancreatic β-cells [154,155,156]. These mechanisms closely overlap with those described in the preceding sections.

PBDs therefore influence T2D risk through several converging pathways rather than through a single glucose-lowering mechanism [40]. Human intervention studies reviewed above show that low-fat or minimally processed PBDs can improve HOMA-IR and other measures of insulin sensitivity, reduce visceral and ectopic lipid accumulation, and improve β-cell responsiveness, particularly when accompanied by reductions in body weight and energy intake [74,112]. Long-term diabetes-prevention studies further demonstrate that sustained improvements in weight, diet quality, and physical activity can markedly reduce progression from prediabetes to T2D [157]. Although these prevention trials were not specifically plant-based interventions, they support the clinical relevance of the metabolic pathways targeted by high-quality PBDs.

#### 3.10.3. Cardiovascular Disease

Obesity promotes CVD through a combination of endothelial dysfunction, atherogenic dyslipidemia, chronic inflammation, oxidative stress, hypertension, and IR [158,159,160,161]. Reduced nitric oxide bioavailability, inflammatory cytokine signaling, altered adipokine secretion, and oxidation of LDL collectively promote vascular dysfunction and atherosclerosis.

PBDs modify several of these pathways concurrently. This indicates that PBDs can improve LDL-cholesterol handling through viscous fibre, phytosterols, and favourable dietary fat quality, while reductions in adiposity and improvements in insulin sensitivity further reduce inflammatory and oxidative stress [162]. PBDs have also been associated with lower circulating markers of vascular inflammation [113,163]. Thus, the cardiovascular benefit of PBDs is likely to reflect combined modification of lipid metabolism, vascular inflammation, adiposity, and metabolic function rather than any single nutrient-specific effect. However, direct mechanistic trials linking whole PBDs with vascular endpoints remain less extensive than evidence for changes in conventional cardiovascular risk factors.

#### 3.10.4. Metabolic Dysfunction-Associated Steatotic Liver Disease

MASLD represents a hepatic manifestation of obesity-related metabolic dysfunction and is closely linked to IR, excess fatty-acid delivery, DNL, and ectopic lipid accumulation [164]. These pathways create a direct mechanistic bridge between adipose-tissue dysfunction and hepatic steatosis.

PBDs influence MASLD primarily through reductions in body weight and ectopic lipid deposition, improvements in insulin sensitivity, and favourable changes in dietary fat quality. In the 16-week low-fat vegan trial discussed above, hepatocellular lipid content decreased substantially alongside improvements in IR, supporting hepatic lipid depletion as a plausible mediator of metabolic benefit [29]. Human overfeeding studies also indicate that saturated fat promotes hepatic fat accumulation more strongly than polyunsaturated fat, suggesting that the fat-quality profile of healthful PBDs is relevant independently of total energy intake [69]. However, because most PBD interventions also reduce spontaneous energy intake and body weight, the extent to which hepatic improvements are attributable specifically to dietary composition remains uncertain.

## 4. Discussion

### 4.1. Principal Findings

Our review synthesized clinical weight-loss evidence and the cellular and molecular mechanisms through which high-quality PBDs influence obesity and its metabolic sequelae. Across the domains examined, a consistent theme emerged: PBDs act not through a single dominant mechanism but by simultaneously modifying several intermingling pathways governing energy balance, adipose tissue biology, insulin action, lipid metabolism, inflammation and gut–host signaling. Their low energy density and high fibre content can reduce spontaneous energy intake without necessarily increasing perceived hunger; reductions in visceral and ectopic lipid are associated with improved insulin sensitivity; and fermentation of plant carbohydrates yields microbial metabolites that influence enteroendocrine signaling and substrate metabolism [61,62,66,67,76,78]. The liver emerged as a particularly responsive target of these pathways. In the 16-week low-fat vegan trial, hepatocellular lipid fell by 34.4%, a change that correlated with reduced IR and was accompanied by a 14.1% rise in the thermic effect of food [29], while reduced visceral adiposity would be expected to lower portal free fatty acid delivery to the liver [67,92]. Together with the more favourable fat profile of healthful PBDs, these findings support hepatic lipid depletion as a potential pathway linking PBDs with improved insulin sensitivity. Human overfeeding studies have shown greater hepatic fat accumulation with saturated fat than with polyunsaturated fat, with saturated fat also increasing circulating ceramides [69,94]. These overlapping effects are consistent with benefits of the dietary pattern as a whole, although the contributions of individual components and concurrent weight loss cannot be separated.

### 4.2. Strength and Limitations of the Human Evidence

The strength of the human evidence differs substantially across mechanistic domains. Randomized trials provide relatively consistent support for reductions in energy intake, body weight, IR, ectopic lipid accumulation, and circulating lipids during low-fat or minimally processed PBD interventions [29,68,162]. By contrast, direct evidence that whole PBDs modify central appetite circuitry, activate brown or beige adipose tissue, or produce weight loss through specific microbiota-mediated pathways remains limited. Much of the mechanistic support in these areas derives from isolated fibres, polyphenols, or experimentally administered microbial metabolites, which can establish biological plausibility but should not be equated with evidence for complete dietary patterns [44,80]. Evidence that whole PBDs directly modulate inflammatory, redox, and epigenetic pathways is similarly constrained, as many studies rely on circulating biomarkers or isolated compounds rather than tissue-level endpoints [139,163]. For MASLD specifically, the evidence base is narrower still. Magnetic resonance spectroscopy data on hepatic fat derive largely from a single trial programme [29], no PBD trial has used liver histology or validated non-invasive fibrosis measures as an endpoint, and in the cited clamp study with a plant-based arm neither the vegan nor the omnivorous high-protein diet improved hepatic insulin sensitivity, indicating that hepatic and peripheral insulin action may respond differently to dietary composition [71]. Whether the reductions in steatosis observed to date translate into resolution of steatohepatitis or regression of fibrosis in MASLD is therefore unknown.

### 4.3. Disentangling Dietary Composition from Energy Balance

A central interpretive challenge is that metabolic improvements during PBD interventions frequently occur alongside reductions in energy intake and body weight, making it difficult to separate effects attributable to dietary composition from those secondary to negative energy balance [45]. Where energy- and weight-matched comparators have been used, plant origin alone has not consistently determined the metabolic response, suggesting that food quality and matrix, rather than the mere exclusion of animal foods, are decisive. This distinction is particularly consequential for the liver, where two compositional features of healthful PBDs may act partly independently of energy balance: the replacement of saturated with unsaturated fat, which, in isocaloric overfeeding, reduced hepatic fat deposition [69,94], and lower exposure to added sugars and refined carbohydrates, which may reduce substrate availability for hepatic DNL [33,34]. Conversely, unhealthful PBDs rich in refined grains and sugar-sweetened beverages may sustain lipogenic drive despite the absence of animal foods. This distinction has direct clinical relevance: healthful PBDs emphasizing whole and minimally processed plant foods confer benefits that are attenuated, or reversed, when the pattern is dominated by refined and ultra-processed plant foods [25].

### 4.4. Clinical and Public Health Implications

Beyond weight reduction, the mechanisms reviewed here connect PBDs to the principal clinical consequences of obesity, including T2D, CVD, MASLD, and several obesity-related cancers [148,149]. Because these conditions share upstream drivers, hyperinsulinemia, chronic inflammation, ectopic lipid, and adipokine imbalance, a dietary strategy across several of them at once has a plausible advantage over single-pathway interventions [99]. MASLD illustrates this convergence most clearly. Because it now affects an estimated 1.3 billion adults, liver-directed pharmacotherapy remains limited, and modest sustained weight loss can be associated with improvement in steatosis and steatohepatitis, a dietary pattern that concurrently reduces energy intake, visceral adiposity, hepatic fat, and saturated-fat exposure is well suited to serve as first-line management and as a foundation on which pharmacological therapy can be layered [9,29,69]. The favourable associations of healthful PBD indices with lower liver cancer risk [152], and the greater attenuation of methylation age among participants who improved MRI-assessed liver fat [146,147], suggest that hepatic benefit may extend across the full disease spectrum from steatosis to hepatocellular carcinoma. The emphasis of PBDs on nutrient-dense, fibre-rich, minimally processed foods also supports their potential as a scalable and equitable population-level strategy, particularly when culturally adaptable and grounded in affordable, accessible foods [165]. At the same time, poorly planned PBDs carry a risk of nutritional inadequacy, and appropriately structured patterns with monitoring where necessary remain important [35].

### 4.5. Limitations of the Review

Our review has several limitations. As a scoping review, it mapped the breadth of mechanistic evidence rather than performing a formal risk-of-bias appraisal or quantitative synthesis. The search was restricted to English-language human studies indexed in two databases, and the heterogeneity of PBD definitions across studies limited direct comparison. The mechanistic framework, while useful for organizing the evidence, imposes structure on data that are frequently cross-sectional and cannot establish the full causal sequence linking diet, mechanism, and clinical outcome.

### 4.6. Future Directions

Future studies should incorporate energy- and weight-matched comparators, objective measurement of food intake and energy expenditure, and tissue-level mechanistic endpoints to determine which pathways directly mediate the metabolic effects of PBDs and which reflect secondary consequences of reduced energy intake. For MASLD, trials should enrol participants with imaging-confirmed steatosis, measure hepatic fat by magnetic resonance and fibrosis by elastography, apply hyperinsulinaemic-euglycaemic clamps to distinguish hepatic from peripheral insulin action, and quantify DNL and gut-derived signals such as SCFAs and endotoxin, so that liver-specific effects of dietary composition can be separated from those of weight loss. Because histological endpoints such as steatohepatitis resolution and fibrosis regression apply to MASH rather than to steatosis alone, dedicated trials in biopsy-confirmed MASH are needed to establish whether PBDs alter disease progression rather than hepatic fat content only. Longer-term trials with clinical endpoints, and studies in diverse populations, are needed to establish whether the mechanistic benefits observed translate into durable, equitable reductions in the burden of obesity and chronic-disease sequelae.

### 4.7. Experimental Longevity Diets and Human Translation

Experimental longevity diets provide context rather than direct evidence for habitual PBD treatment of obesity. In mice fed 25 diets, low-protein, high-carbohydrate patterns were associated with the longest lifespan [166]. Periodic low-calorie, low-protein fasting-mimicking diets also increased median, but not maximum, lifespan in animals [167]. Human translation has been tested in a randomized trial of 100 generally healthy adults: a fasting-mimicking diet low in calories, sugars, and protein and high in unsaturated fats, consumed for five consecutive days per month for three months, reduced body weight and fat, blood pressure, and IGF-1 [168]. These regimens differ from habitual PBDs, and the human findings concern short-term risk markers rather than lifespan. Sustained weight loss, lean-tissue preservation, and long-term clinical outcomes require further exploration in human studies to inform obesity management.

## 5. Conclusions

Obesity arises from interconnected disturbances in energy balance, adipose tissue function, central and peripheral appetite regulation, lipid metabolism, inflammation, and metabolic signaling. The appraised evidence supports that PBDs’ low energy density and high fibre content can facilitate reduced energy intake, while improvements in visceral and ectopic lipid accumulation, insulin sensitivity, and lipid metabolism may further improve metabolic health. Additional effects involving gut microbial diversity, enteroendocrine signaling, intestinal barrier function, and substrate metabolism provide a compelling biological framework, although direct human evidence for several of these mechanisms remains underexplored.

Beyond weight reduction and weight maintenance, high-quality PBDs are associated with lower risks of adiposity-associated T2D, CVD, and selected cancers. The consistent reduction in hepatic fat observed with healthful PBDs, together with their favourable dietary fat quality and low lipogenic substrate load, makes them a rational first-line dietary strategy for MASLD, although histological and fibrosis endpoints remain to be demonstrated. Future rigorously controlled, energy- and weight-matched trials are needed to distinguish cardiometabolic and hepatic effects attributable to dietary composition from those secondary to reduced energy intake.

Overall, high-quality, nutrient-dense, fibre-rich, minimally processed PBDs represent a biologically plausible, multifaceted, scalable, and equitable strategy for obesity management, with the potential to reduce the global burden of obesity and its major chronic disease sequelae.

## Figures and Tables

**Table 1:** Dietary profiles and their composition.

Dietary Profile	Composition and Interpretation
Vegan	Excludes meat, poultry, fish, eggs, and dairy. The label alone does not specify fat content, processing, or overall dietary quality [35].
Low-fat vegan	Vegan diet with an explicit fat restriction. The 16-week trial discussed here prescribed approximately 75% carbohydrate, 15% protein, and 10% fat, using vegetables, grains, legumes, and fruits without added fats [29].
Whole-food plant-based	Emphasizes minimally processed vegetables, fruits, legumes, and whole grains. Animal-food and added-fat restrictions vary; the study prescription determines the exposure [36,37].
Vegetarian	Umbrella term for meat- and fish-excluding patterns; classifications may include vegan, lacto-vegetarian, ovo-vegetarian, and lacto-ovo vegetarian diets [35].
Lacto-ovo vegetarian	Excludes meat, poultry, and fish but includes dairy and eggs; plant-food quality and energy intake vary [35].
Mediterranean	Emphasizes vegetables, fruits, legumes, whole grains, nuts, and olive oil, generally with fish and variable dairy and poultry, while limiting red and processed meat. It is plant-rich but not necessarily vegetarian [38].
Green-Mediterranean	In DIRECT PLUS, added green tea and Mankai to a walnut-supplemented Mediterranean prescription with further restriction of red and processed meat; not equivalent to a vegan diet [39].
Healthful plant-based diet index	Scores greater intake of whole grains, fruits, vegetables, nuts, legumes, and other healthful plant foods more favourably, with lower scores for less-healthful plant and animal foods. It does not require animal-food exclusion [40].
Unhealthful plant-based diet index	Scores greater intake of refined grains, sugar-sweetened drinks, sweets, and other less-healthful plant foods more favourably, with lower scores for healthful plant and animal foods. It is an observational index, not a recommended prescription [40].

## Data Availability

This review is based on published literature; the sources supporting its findings are cited in the article.

## Abbreviations

AGEs	advanced glycation end products
BAT	brown adipose tissue
BMI	body mass index
CCK	cholecystokinin
CRP	C-reactive protein
CVD	cardiovascular disease
DNA	deoxyribonucleic acid
DNL	de novo lipogenesis
GIP	gastric inhibitory polypeptide
GLP-1	glucagon-like peptide 1
GLUT4	glucose transporter type 4
HOMA-IR	homeostatic model assessment of insulin resistance
IGF2	insulin-like growth factor 2
IL-1β	interleukin 1 beta
IL-6	interleukin 6
IR	insulin resistance
IRS	insulin receptor substrate
LDL	low-density lipoprotein
MASLD	metabolic dysfunction-associated steatotic liver disease
NF-κB	nuclear factor kappa B
NLRP3	NOD-like receptor family pyrin domain containing 3
PBDs	plant-based diets
PI3K	phosphoinositide 3-kinase
PRISMA-ScR	Preferred Reporting Items for Systematic Reviews and Meta-Analyses extension for Scoping Reviews
PYY	peptide YY
RAGE	receptor for advanced glycation end products
RNA	ribonucleic acid
ROS	reactive oxygen species
SAT	subcutaneous adipose tissue
SCFAs	short-chain fatty acids
T2D	type 2 diabetes
TLR4	toll-like receptor 4
TNF-α	tumour necrosis factor alpha
UCP1	uncoupling protein 1
VAT	visceral adipose tissue
WAT	white adipose tissue
